# Single-Bud Expression Analysis of Bud Dormancy Factors in Peach

**DOI:** 10.3390/plants12142601

**Published:** 2023-07-10

**Authors:** Ana Puertes, Helin Polat, Luis Andrés Ramón-Núñez, Matilde González, Gema Ancillo, Elena Zuriaga, Gabino Ríos

**Affiliations:** Valencian Institute for Agricultural Research (IVIA), 46113 Valencia, Spain

**Keywords:** flower-bud dormancy, peach (*Prunus persica*), *DORMANCY-ASSOCIATED MADS-BOX* (*DAM*), flowering, gene co-expression

## Abstract

Transcriptomic and gene expression analysis have greatly facilitated the identification and characterization of transcriptional regulatory factors and effectors involved in dormancy progression and other physiological processes orchestrated during bud development in peach and other temperate fruit species. Gene expression measurements are most usually based on average values from several or many individual buds. We have performed single-bud gene analysis in flower buds of peach across dormancy release using amplicons from the master regulatory *DORMANCY-ASSOCIATED MADS-BOX* (*DAM*) factors, several jasmonic acid biosynthetic genes, other genes related to flowering development, cell growth resumption, and abiotic stress tolerance. This analysis provides a close view on gene-specific, single-bud variability throughout the developmental shift from dormant to dormancy-released stages, contributing to the characterization of putative co-expression modules and other regulatory aspects in this particular tissue.

## 1. Introduction

Bud dormancy in perennial species from temperate climates, particularly fruit tree crops, facilitates plant adaptation to a wide range of intensities and durations of the cold period in autumn–winter, with great impact on agricultural yield and plant survival. During the bud dormancy period, cell growth and division are inhibited by intrinsic factors, irrespective of external environmental conditions, to avoid a premature budbreak with harmful effects on meristem viability [1]. Most commonly, bud dormancy is only released after a quantitative exposure to low temperatures, which is highly dependent on the genotype, referred to as the chilling requirement [2]. The quantification of these chilling requirements has been shown by different mathematical models that consider the hourly distribution of temperatures [3,4,5]. After dormancy release, buds enter an ecodormant stage that enables growth resumption and subsequent bud break when environmental conditions become sufficiently warm for a specific time interval. This warm period is known as the heat requirement and is measured in growing degree hours [6]. Both chilling and heat requirements contribute to the phenological plasticity of plant species [7], with a relevant participation in phenological models aimed at predicting the adaptability of fruit crops to warmer winters under different climate change scenarios [8,9].

The wide range of chilling requirements and high dormancy plasticity observed in a clade of Rosaceae species has been related to the presence and dynamism of a group of MADS-box domain transcription factor genes similar to *SHORT VEGETATIVE PHASE* (*SVP*) and *AGAMOUS-LIKE 24* (*AGL24*) flowering regulators from *Arabidopsis thaliana*, named *DORMANCY-ASSOCIATED MADS-BOX* (*DAM*) [10,11]. Interestingly, a genomic deletion comprising four out of the six *DAM* genes arranged in tandem results in the non-dormant phenotype of the peach mutant, *evergrowing* (*evg*) [12]; whereas, gene silencing of the *MdDAM1* gene in apple induces an *evg*-like phenotype [13]. On the contrary, the ectopic expression of Japanese apricot, *PmDAM6*, induces growth cessation followed by bud formation in transgenic poplar and apple [14,15], and *MdoDAMb* overexpression delays budbreak in apple [16]. Moreover, *DAM*-like genes are tightly linked to quantitative trait loci (QTL) associated with dormancy and flowering-time-related traits in many perennial fruit crops [17,18,19,20,21,22]. In some cases, *DAM*-like genes are considered molecular markers of bud dormancy transitions by virtue of their sharp transcriptional down-regulation prior to or concomitant with dormancy release events [23,24,25,26]. Overall, these data support an important role of *DAM*-like genes in regulating seasonal growth and winter dormancy processes [27,28].

Several downstream transcriptional targets have been postulated to mediate the budbreak and cell proliferation effects of *DAM*-like genes. Among them, *FLOWERING LOCUS T* (*FT*)-like genes are down-regulated by *DAM*-like in dormant leafy spurge and pear [29,30] in close analogy with the role of *FT* in flowering initiation in *Arabidopsis* [31] and *FT*-like function in the coordination of reproductive and vegetative growth cycles in poplar [32]. On the other hand, plant hormones are recurrent candidates to mediate the growth repression function of DAM-like genes. *PpDAM1* binds and activates the abscisic acid (ABA) biosynthesis gene, *PpNCED3*, in transient expression assays in pear [33]; and the ectopic expression of *PmDAM6* and *PpeDAM6*, respectively, modifies the content of the hormones, ABA, and cytokinins (CKs) in apple [15] and ABA, gibberellins (GAs), CKs, and jasmonic acid (JA) in plum [34].

In addition to growth and dormancy regulation processes, transcriptional changes in flower buds involve transcripts associated with improved stress tolerance and flowering development genes [35]. Dormant buds deal with water deficit and chilling/freezing winter conditions by stopping growth and activating specific abiotic stress responses, such as the cold responsive (COR) pathway [36], in the frame of a growth–defense trade-off that ensures a proper management of resources. Among other defense mechanisms, dormant buds of peach up-regulate the *STRESS-ASSOCIATED PROTEIN1* (*PpeSAP1*) gene implicated in water loss regulation and cell growth [37]. Concomitant to growth cessation, dormant flower buds of fruit crops stop microspore development in a specific stage prior to male meiosis [38,39] due to the high sensitivity of tapetum activity to low temperature [40]. Thus, microsporogenesis events, including meiosis, tapetum development, and pollen maturation, are postponed until dormancy is released in order to improve pollen viability. Consequently, many tapetum-specific transcripts, such as the ones leading to the synthesis of sporopollenin, an essential component of the outer cell wall of the pollen grain (exine), are concertedly activated shortly after dormancy release and are bona-fide markers of bud dormancy progression [41].

We have selected several genes with an altered expression profile during flower bud dormancy in peach and have performed single-bud gene expression analysis at different stages of bud dormancy progression, with a particular focus on the dormancy transition sample, showing both opened and closed buds in a budbreak-forcing experiment. This approach allows for a better description of gene expression variability across the transition sample and contributes to more detailed knowledge of the process on a single-bud scale.

## 2. Results

### 2.1. Assessment of Bud Dormancy Stages by Budbreak Forcing

**Figure 1:**
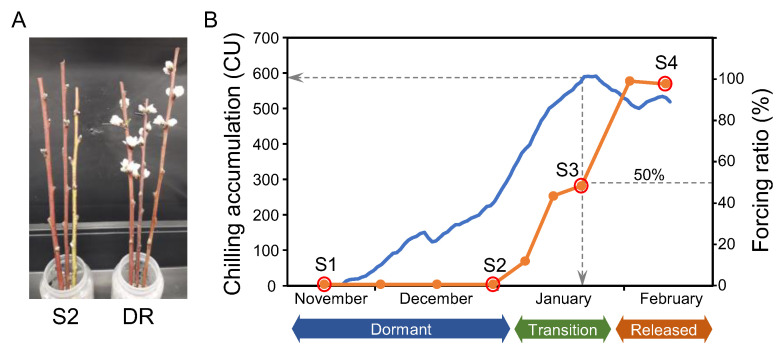
Assessment of dormancy release in peach. (**A**) Detail of a budbreak-forcing assay depicting dormant S2 shoots and shoots from a dormancy-released early cultivar (DR) acting as positive control for budbreak. (**B**) Budbreak-forcing ratio at different times in autumn–winter (orange line) and chilling accumulation (blue line) measured as chilling units (CU). S1, S2, S3, and S4 samples are encircled. Date and chilling accumulation for a forcing ratio of 50% are labelled.

We collected flower buds and shoots from a flat peach cultivar (‘Platibelle’), with intermediate chilling requirements for dormancy release in the autumn–winter period from November to February. In order to assess the dormancy stage of excised shoots, we employed an standard budbreak-forcing assay, with a budbreak percentage of 50% or higher being indicative of dormancy release (Figure 1A), according to previous works [42]. Based on those quantitative budbreak ratios, flower buds representative of early dormant (S1), late dormant (S2), in transition (S3), and dormancy-released stages (S4) were collected individually for subsequent expression analyses. Under the time and environmental conditions of the study, the date of dormancy release was estimated around S3 sample harvest (21 January) with a forcing ratio of 48% after the accumulation of circa 600 CU (Figure 1B). Thus, from the four samples under study, S3 was the only one showing divergent budbreak behaviour, with a closely similar number of opened and closed buds after the forcing assay; whereas, S1, S2, and S4 led to uniformly closed or opened buds.

### 2.2. Single-Bud Analysis of Dormancy-Associated Gene Expression

We selected 13 peach genes, which were previously described to follow a dormancy-dependent expression profile or functionally associated with the bud dormancy process (Table 1). Their relative expression levels were measured in single-bud RNA samples from S1, S2, and S4 (10 buds each) and S3 (71 buds). A higher number of buds was collected in S3 in order to better represent the natural variability present in this transition sample, showing a budbreak ratio close to 50% in forcing experiments (Figure 1B).

**Figure 2:**
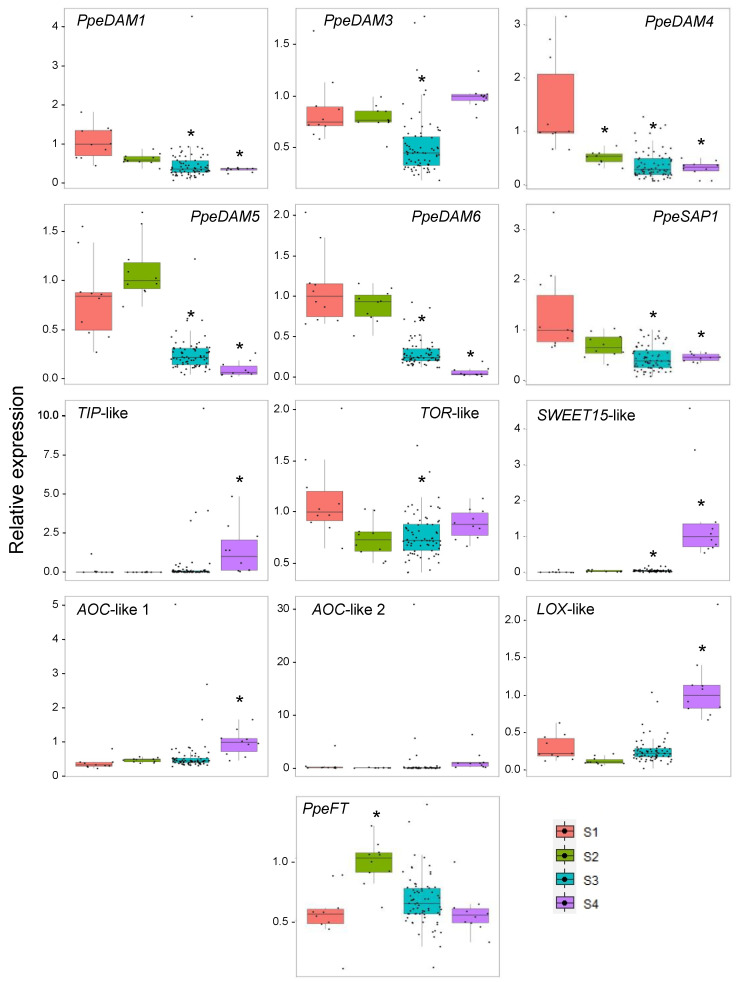
Relative expression of dormancy-associated genes during bud dormancy progression. Single-bud samples are represented by individual points. Box plots of S1, S2, S3 and S4 with different colours according to the legend, with whiskers extending to ±1.5 × IQR (interquartile range). Significant differences with respect to the S1 sample are labelled with an asterisk (*p*-value < 0.001).

From the six *DAM* genes of peach, *PpeDAM2* showed very low expression values under the detectable threshold and consequently, was not included in the analysis. In agreement with previous data, *PpeDAM1*, *PpeDAM4*, *PpeDAM5,* and *PpeDAM6* genes showed decreasing transcript accumulation in transition and dormancy-released samples (S3 and S4) with respect to dormant samples (S1 and S2) (Figure 2, Table S1). For its part, *PpeDAM3* expression did not follow an unequivocal decreasing trend during bud development, being less expressed in S3 than in dormant samples. As expected, *PpeSAP1* was also down-regulated during dormancy progression, in accordance with its proposed role in abiotic stress tolerance. On the contrary, *TONOPLAST INTRINSIC PROTEIN* (*TIP*)-like and *SWEET15*-like genes, associated respectively with cell growth resumption and microsporogenesis pathways, both activated after dormancy release, were strongly up-regulated in S4; whereas, the *TARGET OF RAPAMYCIN* (*TOR*)-like gene slightly increased its transcript amount in S4 after a significant reduction from S1 to S3 (Figure 2, Table S1). The putative JA biosynthesis genes, *ALLENE OXYDE CYCLASE* (*AOC*)-like 1–2 and *LIPOXYGENASE* (LOX)-like, also peaked in the dormancy-released S4, with *LOX*-like showing a second peak in S1. Finally, *PpeFT* showed higher transcript accumulation in S2, being similarly expressed in the remaining samples.

**Figure 3:**
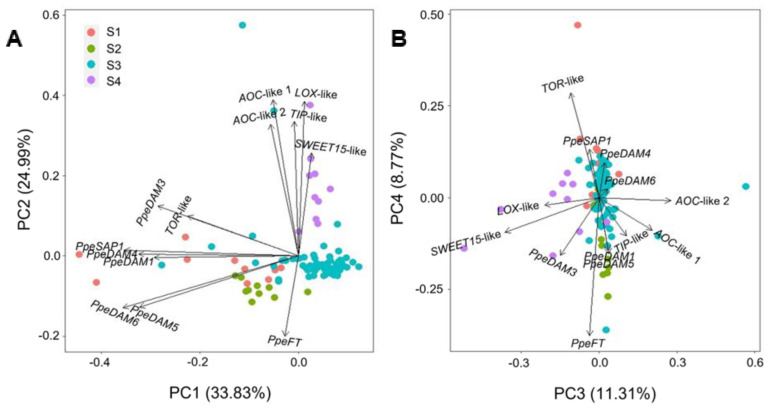
Principal component analysis of gene expression. (**A**) Principal components, PC1 and PC2. (**B**) PC3 and PC4. Single buds are represented by circles with sample-specific colours. The directionality of the contribution of each gene is shown with black arrows. The percentage of the variance explained by the principal components is shown in parentheses.

A principal component analysis (PCA) of expression data led to separated clusters of dormant (S1 and S2) and dormancy-released samples (S4), with transition samples (S3) lying in an approximate intermediate position, and some few individual S3 buds spread out on dormant and dormancy-released groups (Figure 3A). Genes were also grouped by their differential contribution to principal components, PC1 and PC2, with *DAM*-like *PpeSAP1* and *TOR*-like mostly determining PC1, and JA biosynthesis genes, *SWEET15*-like, *TIP*-like, and *PpeFT* contributing to a higher degree to PC2 (Figure 3A). PC3 and PC4 dimensions explained a lower percentage of the variance (8–11%) and were not effectively separating single bud samples in dormant and non-dormant clusters (Figure 3B). Overall, PCA analysis did not help to cluster S3 single buds into two similar populations within selective dormant and non-dormant domains.

**Figure 4:**
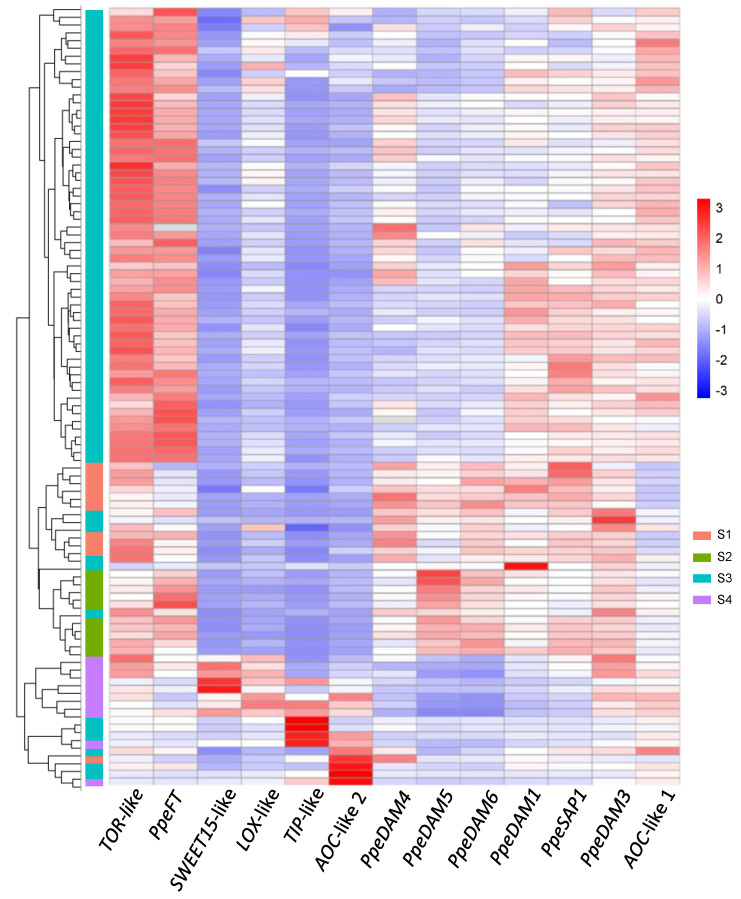
Heat map and clustering of gene expression data. On the *x*-axis, genes under analysis. On the *y*-axis, clustered single-bud samples with their respective dendrogram. S1, S2, S3, and S4 individual samples are labelled with different colours. Gene expression values follow the colour scale shown in the legend.

A heat map and clustering analysis were performed on single-bud gene expression data. The individual buds grouped in specific branches of the dendrogram corresponding to their sampling stage with few exceptions. The majority of S3 buds (59) clustered together, with an additional six buds in the dormant S1 and S2 groups and the remaining six buds in the non-dormant S4 group (Figure 4). In close agreement with PCA and in contrast with the forcing experiment, the clustering and heat map analysis did not split S3 samples into two equivalent parts closer respectively to dormant and non-dormant samples. Intriguingly, one S1 sample clustered in the S4 area in part due to a very high expression level in *AOC*-like 2 gene.

**Figure 5:**
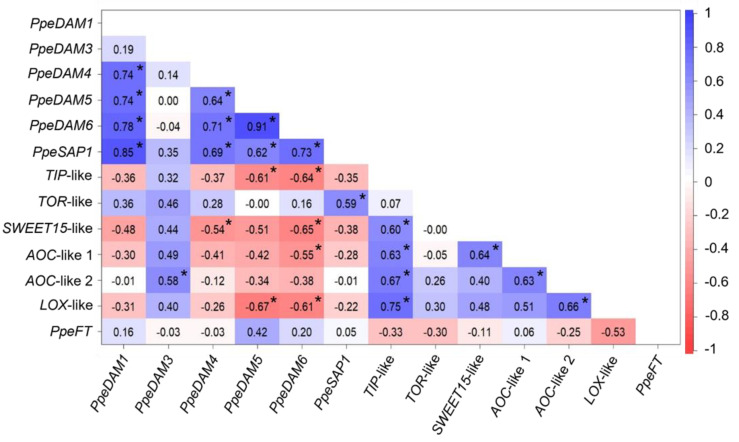
Correlation matrix of gene expression. Each cell in the table shows the Spearman correlation coefficient between two genes. Values between 1 and −1 are coloured following the colour scale shown in the figure. Correlations with a *p*-value < 0.001 in the statistical analysis are labelled with an asterisk.

We analysed paired correlations of single-bud gene expression data from the four time points. With a *p*-value threshold of 0.001, we found a significant positive correlation of *PpeDAM1*, *PpeDAM4*, *PpeDAM5*, *PpeDAM6,* and *PpeSAP1* genes (Figure 5) since these genes are typically highly expressed in dormant buds. Additionally, *AOC*-like 1, *AOC*-like 2, *LOX*-like, *SWEET15*-like, and *TIP*-like showed high positive correlations in most of their combinations, in agreement with their strong up-regulation after dormancy release. In addition, we found significant negative correlation values of *PpeDAM4*, *PpeDAM5,* and *PpeDAM6* with the ecodormancy-related genes, *TIP*-like, *SWEET15*-like, *AOC*-like 1, and *LOX*-like. Surprisingly, we found significant positive correlation values that were not supported by previous research, such as *TOR*-like with *PpeSAP1* and *PpeDAM3* with AOC-like 2 (Figure 5).

### 2.3. Single-Bud Variability in Dormancy-Transition Sample S3

**Figure 6:**
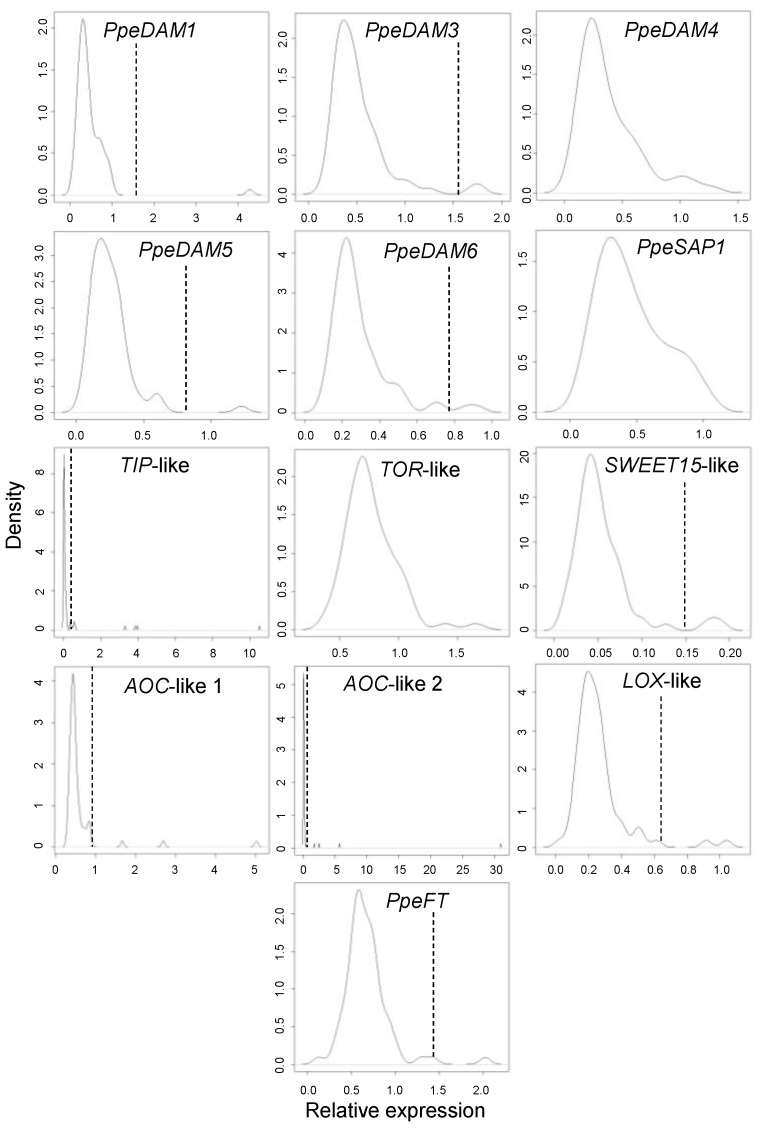
Density graphs of relative expression for each gene in S3 sample. A discontinuous line labels the median plus 3 x IQR value for outlier selection.

We took a more detailed view on the 71 single-bud samples of dormancy-transition S3 in order to avoid the effects of varying environmental conditions of S1, S2, and S4 time points on the distribution and variability of single-bud expression measurements. The density plot shown in Figure 6 highlights the expression value distribution of S3 single buds for the different genes. None of the genes showed a bimodal expression pattern accounting for the approximately equal ratio of opened and closed buds found in forcing experiments (Figure 1B). Although evident shoulder peaks were observed in *PpeDAM1*, *PpeSAP1,* and *AOC*-like 1, among other less pronounced ones, a bimodal expression profile was ruled out in the genes under study by using the “diptest” package in R (Table S2). In addition, buds with unusually high expression values were observed in *PpeDAM1*, *TIP*-like, *AOC*-like 1, *AOC*-like 2, and other genes to a lesser extent. Outlier samples with an expression value higher than the third quartile (Q3) plus three times the interquartile range (IQR) are labelled in Figure 6.

**Figure 7:**
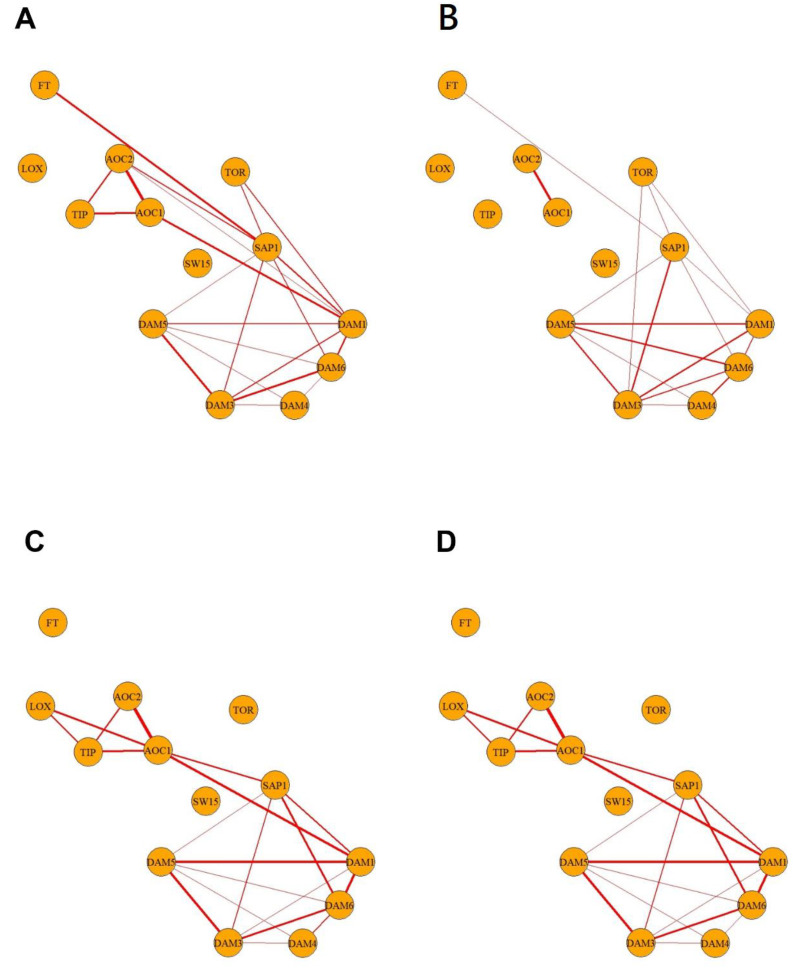
Gene expression correlations in dormancy-transition sample S3. Connections between genes in the graph are indicators of a positive (red line) correlation with *p*-value < 0.001. The value of the correlation coefficient is indicated by the thickness of the line, being a thicker line indicative of a higher correlation coefficient. (**A**) Correlations using expression gene data of individual buds; (**B**) correlations using expression gene data of individual samples but removing the outliers with value higher than Q3 + 3 × IQR; (**C**) correlations using expression gene data of in silico artificial samples; (**D**) correlations using expression gene data of in silico artificial samples without outliers. Gene nomenclature has been shortened for practical reasons to make this figure. Particularly, *SWEET15*-like has been labelled as SW15.

Gene expression in S3 is supposedly devoid of temperature, light, climatic, and dormancy progression bias caused by bud collection at different dates. Thus, correlations in S3 individual buds might highlight genes sharing a common expression pathway or direct transcriptional targets of a given transcription factor. Gene expression correlations in S3 with a *p*-value lower than 0.001 are shown in Figure 7A. Two well-defined clusters of expression were found: *DAM*s, *PpeSAP1*, and *TOR*-like constituted the first one, and JA biosynthesis genes and *TIP*-like led to the second one, with *PpeFT* apparently showing a positive correlation with *PpeSAP1*-like. After removing the outliers according to above conditions (Q3 + 3 × IQR), the analysis of correlations showed few relevant changes. *AOC*-like 1 and 2 lost their previous interactions with *TIP*-like and the *DAM*/*PpeSAP1* cluster (Figure 7B).

To investigate whether the results obtained with individual buds differed from those obtained with pooled buds, we designed an in silico study. This study involved the simulation of RNA isolation of a pool of five bud samples in the S3 phase and subsequent RT-qPCR analysis. For this, we took the average expression values of the different genes studied in five randomly selected samples in the S3 phase. This operation was repeated 71 times, simulating the sample size of individual bud samples. These in silico studies were performed one thousand times, and the subsequent comparations were performed. Based on the results, if more than 80% of the simulations yielded statistically significant outcomes under a *p*-value of 0.001, it was considered that the comparison was significant and is shown in Figure 7D. A similar approach was performed on the S3 devoid of outliers (Figure 7D). In silico pooled samples showed the major two clusters of concerted expression also observed in single-buds, but some interactions of *PpeFT* and *TOR*-like were lost, and the effect of outlier removal was buffered under the pooling strategy (Figure 7C,D).

## 3. Discussion

Transcriptional regulation during bud dormancy progression has been widely studied in perennial plants due to its expected impact on plant adaptation and crop performance under the threat of climate change, leading to a set of differentially expressed genes with regulatory and effector roles in plant dormancy and other bud-specific processes. In peach, *DAM* genes are MADS-box involved in dormancy regulation that, by virtue of their strong dormancy-dependent regulation, have been proposed as expression markers of the dormancy stage [45]. In this work, we have shown that with gene-specific particularities, *PpeDAM1*, *PpeDAM4*, *PpeDAM5,* and *PpeDAM6* are down-regulated concomitantly with bud dormancy progression and release from S1 to S4. This closely agrees with local genomic variations in the chromatin modification, H3K27me3, across *PpeDAM1*, *PpeDAM4*, *PpeDAM5,* and *PpeDAM6* genes [46]. Interestingly, H3K27me3 and/or other chromatin histone modifications (H3K4me3 and H3 acetylation) have been found associated with the transcriptional activity of *DAM*-like genes in different dormancy stages in leafy spurge [47], peach [42,48], pear [49], sweet cherry [50], and apple [51], suggesting the involvement of epigenetic mechanisms in the quantitative chilling-dependent regulation of bud dormancy [28,52,53]. For its part, *PpeDAM3* expression decreased in the transition sample to later increase after dormancy release, in accordance with previous observations by Li et al. [23].

The ectopic expression of *PpeDAM6* in plum increases the expression of several genes of the JA biosynthetic pathway, according to Lloret et al. [34]. In our single-bud assay, we analysed the expression of three genes within this pathway (*AOC*-like 1, *AOC*-like 2, and *LOX*-like), confirming their up-regulation in dormancy-released buds (S4). This increase has been related to the role of JA in anther development and pollen maturation [54], a process initiated in *Prunus* species just after dormancy release in flower buds [55], most likely due to the cold sensitivity of tapetum-dependent activity in pollen development [56]. Thus, one of the most prominent roles of bud dormancy could be to preserve pollen viability and fertility across the winter low-temperature period. Moreover, a decrease in *LOX*-like expression was observed from early dormant (S1) to late dormant (S2) and transition buds (S3), consistent with seasonal JA changes in flower buds of peach [34].

On the other side, the dormancy-dependent expression of *PpeSAP1*, *TIP*-like, and *SWEET15*-like fitted well with their respective proposed function in water retention, cell expansion, and pollen maturation processes [37,41]. However, *TOR*-like did not show a pattern of expression opposite to *PpeSAP1* as published [37], suggesting the presence of complex genetic and/or environmental factors modulating its expression.

Different *FT*-like genes have been proposed to interact with *DAM*-like genes at the transcriptional level. DAM-like proteins bind the promoter of *FT2* gene by chromatin immunoprecipitation experiments in leafy spurge [29], and *FT*-like promoter activity is repressed by several *DAM*-like genes by dual luciferase transient expression assays in pear [30]; whereas two *DAM*/*SVP*-like genes are down-regulated when overexpressing *VvFT* gene in grapevine [57]. We have included in our study the putative ortholog of *Arabidopsis FT* according to reciprocal blastp analysis (*PpeFT*). *PpeFT* did not follow an expression pattern complementary to *DAM* genes, as suggested in other species [29]. In addition, neither all the samples nor the transition S3 showed any observable correlation between *PpeFT* and *DAM* genes. Thus, in our hands, *PpeFT* was not a candidate target of *DAM* regulatory function, although additional analyses on different cultivars, seasons, and other related *FT*-like genes should be performed in order to rule out the interaction.

In this work, we aimed at using transition S3 single buds for studying expression correlations between regulators and targets and between genes belonging to a common bud developmental pathway since this sample is devoid of environmental noise due to different collection dates and S3 single buds show a heterogeneous behaviour regarding dormancy and cell activity. This has been useful to gather evidence supporting the concerted regulation of JA biosynthesis genes (*AOC*-like 1, *AOC*-like 2, and *LOX*-like) and the *PpeDAM*/*PpeSAP1* module. In addition, the S3 sample revealed previously unexpected correlations between genes involved in non-related processes and even showed opposite expression trends during dormancy progression, such as *AOC*-like 1, *AOC*-like 2, and *TOR*-like with the *PpeDAM*/*PpeSAP1* cluster, which was only partially dependent on the presence of outliers. This could be due to single-bud differences in cell activity, sanitary status, or abiotic stresses leading to broad effects on gene expression or alternatively could respond to the function of specific regulatory factors affecting the expression of these transcripts. In any case, the presence of numerous outliers with extremely high expression values suggests that buds establish highly heterogeneous populations at the transcriptional level.

The in silico model performs a simulation of the results that we would have obtained if we had followed a strategy of RNA extraction from sample pools but considering an unusually high number of samples for a gene expression experiment. We can observe, on the one hand, that the two major groups of concerted expression are common in the single-bud experiment and the multi-bud in silico experiment. On the other hand, some gene correlations not belonging to the two major groups were lost in the in silico multi-bud experiment. When we average the gene expression in the in silico multi-bud experiment, the biological variability present in each individual sample may be attenuated. Therefore, some significative correlations between genes in individual samples are not statistically significant in pooled samples, but, interestingly, *LOX*-like interactions are more pronounced under in silico pooling. Both gene expression measurements in individual samples and pooled samples are valid with some slight differences. In fact, gene expression measurements in pooled samples can be useful in reducing technical variability and providing a more global view of gene expression in a group of samples. When we removed the outliers and repeated the in silico multi-bud experiment, we observed very similar correlations in gene expression levels (Figure 7C,D) because of attenuation of individual variation in both cases. In any case, the correlation between the expression levels of different genes suggests that the genes are co-regulated or functionally related. Overall, the standard bud-pooling experimental strategy for expression studies should fulfil most of dormancy-researcher interests, being single-bud analyses recommendable for the testing of transcriptional correlations devoid of environmental interferences.

Our current data showing the expression variability in S3 sample do not support an on/off switch model for the function of *DAM* genes to determine single-bud dormancy release at different times, but we cannot rule out the possibility that additional data, genes, conditions, and scales could affect this hypothesis.

## 4. Materials and Methods

### 4.1. Plant Material

Ten-year-old peach trees (*Prunus persica* L. Batsch cv ‘Platibelle’), grafted onto the rootstock GF677 and grown under standard agricultural practices, were located in Pobla del Duc (Spain) (38° 54′ 11″ N 0° 25′ 38″ W). Shoots for the forcing assay and flower buds for gene expression analyses were collected on the following dates in autumn–winter (2020–2021): 18 November (S1), 2 December, 16 December, 30 December (S2), 7 January, 14 January, 21 January (S3), 2 February, and 11 February (S4).

### 4.2. Dormancy Assessment

Bud dormancy was assessed by the budbreak-forcing assay. For this procedure, twelve young shoots of about 20 cm were collected per date from different trees. We left 5–6 flower buds per shoot. After cutting the basal and the apical ends of shoots, they were distributed in groups of three in glass bottles containing tap water and incubated in a phytotron set at 24 °C, with a 16 h:8 h light:dark cycle. Water was renewed three times per week. Buds reaching the green stage according to the Baggiolini code [58] after 14 days incubation were considered opened. Chilling accumulation was estimated following the Utah model [4].

### 4.3. Single Bud RNA Isolation and qRT-PCR

Total RNA of peach single buds was isolated using the Plant/Fungi Total RNA Purification Kit (Norgen, Thorold). To improve the extraction process, polyvinylpyrrolidone (PVP-40) was added at a concentration of 1% (*w*/*v*) to the kit extraction buffer prior to use. The procedure of removing contaminant genomic DNA was also carried out during the isolation. About 500 ng of RNA was reverse transcribed with PrimeScript RT reagent kit (Takara Bio, Otsu, Japan). Quantitative RT-PCR was performed on a StepOnePlus Real-Time PCR System (Life Technologies, Carlsbad, CA, USA) with SYBR premix Ex Taq (Tli RNaseH plus) (Takara Bio), using 2 μL diluted (10× or 20×, according to RNA concentration) first-strand cDNA, and a final volume of 20 μL. PCR conditions were 10 min at 95 °C, followed by 40 cycles of 15 s at 95 °C, and 1 min at 60 °C. The presence of a single peak in the dissociation curve after PCR and the size estimation of the amplified product by electrophoresis was used for evaluating the specificity of the amplification.

SAND-like was used as the reference gene for the analysis [37,59]. Relative expression was measured using a relative standard curve. Results were the average of 2–3 technical replicates each. Primers used in this study are listed in Table S3.

### 4.4. Statistical and Bioinformatic Analysis

For statistical analysis and graphs, we used the open-source programming language and environment R (version 4.2.2) through the “RStudio” interface (version 2022.12.0 + 353). All expression values were recalculated dividing by the highest median of the stages for each gene. To compare the expression levels of a gene between two groups of samples, we performed the non-parametric Mann–Whitney U test using the R function “wilcox.test()” from the package “stats”

For box plots, we used the package “ggplot2” and the function “ggplot”. Single-bud points were shown with “geom_jitter()”. PCA figure was made using the package “ggfortify” and the function “autoplot”, built from a correlation matrix. It was standardized to get a median of 0 and a standard deviation of 1. For the gene expression correlation matrix, we used the package “psych” and the function “corPlot”. In density graphs, the function “plot(density())” was used. Outliers were removed from the analysis following Tukey’s fences, being k parameter equal to 3.

We used the function “pheatmap” to draw clustered heatmaps with scaled gene expression (using value “row” in the argument “scale”). The function also aggregates the rows using kmeans clustering.

To calculate the Spearman correlation between the expression levels of two genes, we utilized the R function “cor.test()” with the “spearman” method. In order to prevent overrepresentation of the S3 phase, we randomly selected only 10 samples from the S3 phase while including all samples from phases S1, S2, and S4. This process was simulated a thousand times, and the average Spearman coefficient correlation was used to construct the gene expression correlation matrix. We considered correlations with a *p*-value < 0.001 in over 80% of the simulations as statistically significant. For the unimodality/multimodality assessment, we used Hartigans’ dip test with the function “diptest”. For the in silico multi-bud experiment, mean gene expression values of five individual random dormancy-transition buds S3 were used to produce an artificial multi-bud sample for each gene. This process was repeated 71 times to produce an artificial pool of multi-bud dormancy transition sample S3. These artificial S3 samples were compared versus the S1, S2, and S4 samples using the Mann–Whitney U test. Furthermore, these artificial S3 samples were used to perform a Spearman correlation between the 13 genes under study. Due to the limited number of possible combinations in S1, S2, and S4 phases, this simulation was only performed in the S3 phase. Therefore, comparisons in the in silico studies are made using the artificial pool of samples in phase S3 and individual buds in phases S1, S2, and S4.

This experiment and the subsequent statistical analysis were repeated 1000 times. Correlations between the 13 genes in the artificial group S3 were considered significant when more than 80% of them had a *p*-value < 0.001. Furthermore, we repeated the same experiment and the statistical analysis without the outliers. For the correlation diagrams, we used the package “igraph” and the function “plot(graph_from_data_frame())”.

## Figures and Tables

**Table 1:** List of genes used in this work.

Name	Transcript	Protein ID	Description	Process	Peach Reference
*PpeDAM1*	Prupe.1G531100	ABJ96361	MADS-box transcription factor	Bud dormancy regulation	[23]
*PpeDAM3*	Prupe.1G531400	ABJ96364	MADS-box transcription factor	Bud dormancy regulation	[23]
*PpeDAM4*	Prupe.1G531500	ABJ96358	MADS-box transcription factor	Bud dormancy regulation	[23]
*PpeDAM5*	Prupe.1G531600	ABJ96359	MADS-box transcription factor	Bud dormancy regulation	[23]
*PpeDAM6*	Prupe.1G531700	ABJ96360	MADS-box transcription factor	Bud dormancy regulation	[23]
*PpeSAP1*	Prupe.2G010400	XP_007218502	A20/AN1 Zn finger	Abiotic stress	[37]
*TIP*-like	Prupe.2G229500	XP_007218847	Aquaporin	Water movement ^(^*^)^	[37]
*TOR*-like	Prupe.8G151300	XP_020425391	Protein kinase	Cell growth ^(^*^)^	[37]
*SWEET15*-like	Prupe.1G220700	XP_007222479	Sugar transporter	Pollen maturation ^(^*^)^	[41]
*AOC*-like 1	Prupe.1G306100	XP_007223720	Allene oxide cyclase	Jasmonic acid biosynthesis ^(^*^)^	[34]
*AOC*-like 2	Prupe.3G239900	XP_00721504	Allene oxide cyclase	Jasmonic acid biosynthesis ^(^*^)^	[34,43]
*LOX*-like	Prupe.2G005300	XP_007220253	Lipoxygenase	Jasmonic acid biosynthesis ^(^*^)^	[34]
*PpeFT*	Prupe.6G364900	ACH73165	Phosphatidylethanolamine-binding protein	Flowering initiation	[44]

^(^*^)^ Based on protein similarity.

## Data Availability

Data recorded in the current study are available in all tables and figures of the manuscript.

## Supplementary Materials

The following supporting information can be downloaded at: https://www.mdpi.com/article/10.3390/plants12142601/s1, Table S1. Statistical differences in gene expression levels of various genes between samples using the Mann–Whitney U test; Table S2. Hartigan’s test for assessing bimodality in S3 samples using the R package ‘diptest’; Table S3. Primers used in this study [60].
