# Supplementary material for: Single-Bud Expression Analysis of Bud Dormancy Factors in Peach
Source: Plants (Basel). 2023 Jul 10;12(14):2601. doi: 10.3390/plants12142601 (PMC10385799; doi:10.3390/plants12142601)
Supplement: Supplementary file 1 [file plants-12-02601-s001.zip › plants-2457135-supplementary.pdf]

Table S1. Statistical differences in gene expression levels of various genes between samples using the Mann-Whitney U test. The upper table presents the p-values obtained using individual buds. The lower table shows the number of experiments with significant differences after pooling S3 samples using different p-values as cut-offs.

INDIVIDUAL BUDS

**Comparations**	*PpeDAM1*	*PpeDAM3*	*PpeDAM4*	*PpeDAM5*	*PpeDAM6*	*PpeSAP1*	*TIP*-like	*TOR*-like	*SWEET15*-like	*AOC*-like 1	*AOC*-like 2	*LOX*-like	*PpeFT*
**S1 vs S2**	0,0115	0,8534	4,3E-05	0,0232	0,4359	0,0232	0,5288	0,0052	0,0015	0,0052	0,1431	0,0015	1,3E-04
**S1 vs S3**	4,4E-05	1,3E-04	4,1E-06	9,5E-06	8,2E-07	1,5E-05	0,0127	0,0009	8,1E-05	0,0017	0,0590	0,5903	0,1178
**S1 vs S4**	1,1E-05	0,0288	1,8E-04	1,1E-05	1,1E-05	1,1E-05	3,2E-04	0,1051	1,1E-05	7,6E-05	0,0089	1,1E-05	0,9118
**S2 vs S3**	0,0084	1,2E-04	0,0098	6,5E-07	1,0E-06	0,0060	4,7E-05	0,7143	0,1704	0,6204	0,6824	1,0E-04	8,8E-05
**S2 vs S4**	2,1E-04	0,0007	0,0046	1,1E-05	1,1E-05	0,0115	1,1E-05	0,0892	1,1E-05	4,9E-04	4,3E-05	1,1E-05	2,1E-04
**S3 vs S4**	0,6719	1,2E-05	0,9362	1,7E-04	1,2E-06	0,3696	2,2E-04	0,0286	3,6E-07	9,7E-05	8,3E-06	7,6E-07	0,0636

p < 0,05
p < 0,01
p < 0,001

ARTIFICIAL POOLED BUDS

**Comparations**	*PpeDAM1*	*PpeDAM3*	*PpeDAM4*	*PpeDAM5*	*PpeDAM6*	*PpeSAP1*	*TIP*-like	*TOR*-like	*SWEET15*-like	*AOC*-like 1	*AOC*-like 2	*LOX*-like	*PpeFT*
**S1 vs pool S3**	999	1000	1000	1000	1000	1000	998	1000	1000	1000	0	0	999
**S2 vs pool S3**	979	1000	941	1000	1000	999	1000	0	973	17	241	1000	1000
**pool S3 vs S4**	958	1000	4	1000	1000	1	976	982	1000	997	989	1000	993

Table S2. Hartigan's test for assessing bimodality in S3 samples using the R package 'diptest'. The p value associated to the Hartigan's test for each gene is shown.

Genes	Hartigans' dip test p-value
PpeDAM1	0.9908
PpeDAM3	0.8038
PpeDAM4	0.9612
PpeDAM5	0.8157
PpeDAM6	0.988
PpeSAP1	0.9902
TIP-like	0.9819
TOR-like	0.9461
SWEET15-like	0.9868
AOC-like 1	0.9858
AOC-like 2	0.9686
LOX-like	0.9831
PpeFT	0.9228

Table S3. Primers used in this study.

Gene	forward	reverse	Reference
SAND-like	TCGTGGGTACCAGGAAAACGACAT	CCTGCTAGCTTGTGTTCATCTCCA	[37]
PpeDAM1	GGGGACGATGAAAATGACGAGGGAG	GTGGTGGAGGTAGCAGATTCAGAT	[60]
PpeDAM3	ACCAGCTAAGGCAGACGATGA	GAGGGAGAGAGACTGAGAGCA	[60]
PpeDAM4	TGTGGCACTTGAGAAAAAGGGA	CAGGTTACTTTCCCCAGGCCAC	[60]
PpeDAM5	CCCCGAAACCCACGAACGAAGATG	CAGCACTGTTGCAGGTGGTG	[60]
PpeDAM6	CCAACAACCAGTTAAGGCAGAAGA	GGAAGCCCCAGTTTGAGAGA	[24]
PpeSAP1	ACACAGGCTTCCTCTACTCCATCTTT	GAACCCTCATTCCGAGACATTTATCAG	[37]
TIP-like	AGTTGTTGCTTGCTTGCTACTCAAG	GCACCAACAATCAAACCAATTGCGA	[37]
TOR-like	GCAGTACCAAAGAAGATTGGGCAGA	GCAAATAACTCGCGCCCAACAAAT	[37]
SWEET15-like	TTCCAATCGGTGCCATATCTGGTAG	GAAGAGCCCCACGTTCATAAAACCA	[60]
AOC-like 1	ACAAGATCTACTCTGGGGACTTGG	AAGCTGTAGACTGCCTCGTACCTG	This work
AOC-like 2	GTTAATTCTCTTGAAGACCTCGTC	GAAGCTGTAAATAGCCTCATATCG	This work
LOX-like	CAGTGTCCACGGTGACCACAA	CGCTAACAAGCTCCAAAAGAAGTG	This work
PpeFT	GGAGGTTAACAATGGTTGCGAG	TCCTTAAGGTTGGGGTCACTTG	This work
